# Erbin Inhibited Angiogenesis *in vitro* with the Inhibition on the STAT3 Pathway in Breast Cancer Cells

**DOI:** 10.2174/0109298673366195250331175911

**Published:** 2025-04-23

**Authors:** MingZhen Zhao, HaiLan Xu, Yu Sun, XinYang Li, LingLing Wang, Xing Zhao, Die Mu, YaLei Li, LiXin Sun

**Affiliations:** 1 Affiliated Hospital of Chengde Medical University, 067000, Chengde, Hebei, China

**Keywords:** Erbin, angiogenesis, STAT3, breast cancer, HUVECs, VEGF

## Abstract

**Background:**

Angiogenesis plays an important role in progression of tumors including breast cancer, which accounts for the vast majority of women's malignant tumors globally, to meet the excessive requirement of oxygen and nutrition for growth, metastasis, and invasion of the tumor. Therefore, targeting tumor angiogenesis has turned into a significant target for cancer therapy. Erbin has a significant effect on the initiation and progression of cancer, including breast cancer, but its role in inhibiting vascular endothelial cell proliferation and angiogenesis by breast cancer cells remains unclear.

**Methods:**

In this study, human SKBR3 and MCF-7 breast cancer cells were used and transfected with the plasmid and siRNA for overexpression and silence of Erbin, respectively. Western blot, qRT-PCR, CLEIA, CCK-8 and Matrigel Tube Formation Assay were used for the proteins detection, mRNAs detection, detection of VEGF in the culture supernatants, detection of cell proliferation and detection of the angiogenic ability of HUVECs *in vitro*, respectively.

**Results:**

It was shown that the expression of both Erbin protein and mRNA in SKBR3 cells was lower compared to that in MCF-7 cells (*p* < 0.05). While the expression of VEGF protein was higher in SKBR3 cells than that in MCF-7 cells (*p* < 0.05). Furthermore, the VEGF protein and mRNA in the cells, VEGF protein in the culture supernatant, HUVEC proliferation in the conditioned medium at 16 h and 24 h, the total length of tube formation in the conditioned medium, and pSTAT3 protein in the cells, were downregulated by transfection of Erbin gene in SKBR3 cells and upregulated (excluding HUVEC proliferation at 16 h) by transfection of Erbin siRNA in MCF-7 cells compared with their NC cells (*p* < 0.05).

**Conclusion:**

It can be concluded that Erbin, with inhibiting the STAT3 pathway, suppresses the proangiogenic effects of breast cancer cells, thereby suggesting its potential as a therapeutic target for breast cancer.

## 1 INTRODUCTION

Breast cancer is the most prevalent type of malignant tumor among women globally [1, 2]. In recent years, there has been a substantial rise in its incidence, coupled with a trend towards younger patient demographics,which not only imposes a profound social and economic burden but also severely disrupts the health and quality of life for affected women [3, 4]. Even if remarkable progress has been achieved in breast cancer therapy, such as targeted therapy, hormone therapy, surgery, chemotherapy and radiotherapy, breast cancer continues to be the leading cause of cancer-related deaths among women [5-8]. The origination and progression of breast cancer undergo a multistep process, and angiogenesis is a critical process in the proliferation and metastasis of cancer cells [9, 10] including breast cancer [11-13].

Vascular endothelial cell proliferation is essential for angiogenesis which plays an important role in cancer. One of the characteristics of cancer is the dysregulated function of angiogenesis, in which, with uncontrolled regulation in the body, the blood vessels are newly formed to meet the requirement of oxygen and nutrition for growth, metastasis, and invasion of the tumor [14-16]. There are pro-angiogenic factors in the tumor microenvironment including vascular endothelial growth factor (VEGF), basic fibroblast growth factor (bFGF), platelet-derived growth factor (PDGF). Exposure of these pro-angiogenic factors to host mature vascular endothelial cells result in angiogenesis [17-19]. Under the action of exposure to these angiogenic factors and chemokines in tumor tissue, new collateral blood vessels are formed in order to supply more nutrients to the tumor tissue [20]. In addition, at the tumor site, endothelial progenitor cells (EPCs) are recruited. EPCs refer to endothelial precursors that are differentiated from bone marrow and hematopoietic cells [21]. They are attracted by growth factors, cytokines, and hypoxia-related signaling pathways. Once there, they differentiate into mature endothelial cells, and a vascular channel forms by endothelial cells clumping together, division, and proliferation, based on the stimulation of angiogrowth factor, to provide enough nutrients to tumor cells [22].

Targeting tumor angiogenesis has turned into a significant target for cancer therapy, since angiogenesis plays an important role in tumor progression. The success in practical application of targeted drugs including bevacizumab, Sorafenib, and Sunitinib in clinical practice advocates the successful utilization of anti-angiogenic therapy in cancer treatment [23-26]. Poor therapeutic effects of these drugs, however, have been reported on certain patients and promotional effects on tumor progression have even been found [27-31]. Therefore, new target for anti-angiogenic therapy is required for tumor therapy such as breast cancer therapy.

One of the powerful mediators of angiogenesis is VEGF which plays important roles in tumor growth, in addition to a variety of processes including physiological processes in healthy humans such as embryonic development and wound healing [32-34]. It is important in cancer because VEGF induces proliferation, migration, and tube formation of endothelial cells to form new blood vessels [35-38]. VEGF expression can be up-regulated by activation of STAT3 because STAT3 directly binds to the promoter of VEGF [39-42]. Suppressing STAT3 to inhibit neo-angiogenesis could be a promising strategy for preventing or delaying the formation of new tumors in cancer therapy [43-45]. Inhibition on vascular endothelial cell proliferation strongly contributes to angiogenesis inhibition.

Erbin (Erbb2 interacting protein), a type of adaptor protein in the LAP protein family, can directly bind to the C terminus of Her2 resulting in targeting of basolateral localization of the Her2 receptor [46, 47], inhibit Ras-Raf-ERK signaling pathway which repress mitogen-activated protein kinase (MAPK), inhibit nuclear factor-κB (NF-κB) signaling pathway and transforming growth factor β (TGF-β) signaling pathway [48]. Erbin plays an important role in initiation and progression of cancer including breast cancer [49-51], however, its role in downregulation of VEGF in breast cancer cells to inhibit vascular endothelial cell proliferation and angiogenesis remains unclear. In this study, conditioned medium from SKBR3 and MCF-7 breast cancer cells with and without Erbin upregulation or downregulation by Erbin gene or Erbin siRNA transfection, respectively, was used to detect the inhibitory role of Erbin in HUVEC proliferation and angiogenic effect.

## 2 MATERIALS AND METHODS

### 2.1 Cell Culture and Transfection

The human SKBR3 and MCF-7 breast cancer cells (Wuhan Pricella Biotechnology Co., Ltd., Wuhan, China) were cultured in DMEM and RPMI 1640 medium, respectively, and the HUVEC (Shanghai EK-Bioscience Biotechnology Co., Ltd, Shanghai, China) was cultured in DMEM medium, in a humidified atmosphere of 5% CO_2_ at 37°C. The 10% fetal bovine serum (FBS) (VivaCell, Shanghai, China) was supplemented into the medium. The SKBR3 cells at a density of 6×10^5^ cells/well were seeded into six-well culture plates, and after an overnight culture, the plasmids encoding the full-length Erbin were transfected into the SKBR3 cells using the Lipofectamine 3000 Reagent (Thermo Fisher Scientific, Waltham, MA USA) in accordance with the manufacturer's instructions. After 36h culture for Erbin over-expression, the transfected cells were collected for further assay. The replacement of the plasmids with Erbin siRNA was used on MCF-7 cells after an overnight culture of the cells seeded at a density of 6 × 10^5^ cells/well into six-well culture plates for Erbin siRNA transfection using the Lipofectamine 3000 Reagent (Thermo Fisher Scientific, Waltham, MA USA) in accordance with the manufacturer's instructions followed by 36h culture of the cells for Erbin silence, and the transfected cells were collected for further assay. The 1% FBS in the medium was used for transfection. The non-targeting controls (NC) for the plasmids and siRNA were used.

### 2.2 Western Blot Assay

The total protein was extracted from the cells with RIPA Lysis Buffer (Zhongshi Gene Technology Co., Ltd., Tianjin, China) which contains an inhibitor cocktail of protease and phosphatase, and measured with BCA protein assay kit (Applygen Technologies Inc., Beijing, China) to determine the concentration of the protein. SDS-PAGE with equal amounts of the proteins subjected to it was performed before electro-transference to a polyvinylidene difluoride (PVDF) membrane (Roche, Mannheim, Germany). Non-specific binding was blocked by two hours of the membranes in PBS-Tween-20 with 5% nonfat milk (Solarbio, Beijing, China) in it and the primary antibodies, anti-Erbin (Affinity Biosciences, Jiangsu, China), anti-VEGF, anti-STAT3 (Huabio, Hangzhou, China) and anti-pSTAT3 (HUABIO, Zhejiang, China), were used in the overnight incubation of the membranes at 4°C. The membranes were washed 3 times with Tris-buffered saline and Tween 20 (TBS-T) and incubated with corresponding secondary antibodies. The protein bands were visualized using chemiluminescence kit (Proteintech, USA) and quantified using the ImageJ software (NIH, Bethesda, MD, USA).

### 2.3 qRT-PCR Assay

The total RNA in the cells was extracted using Trizol reagent (Invitrogen, Carlsbad, USA) and was reverse transcribed into cDNA using FastQuant RTkit (Tiangen Biotech Co., Ltd., Beijing, China), in accordance with the manufacturer's instructions. The sequences of target genes and reference gene were amplified by quantitative reverse-transcriptase polymerase chain reaction (qRT-PCR) using SuperReal PreMix Plus kit (Tiangen Biotech Co., Ltd., Beijing, China) and quantified by calculation of the 2^-ΔΔct^, based on and an internal reference gene, β-actin. The primers were used as follows: Erbin: Forward Sequence GTCAAGACACCTCACTCTGCTC, Reverse Sequence CATGAGCTTTGAACTTTTCCTCTG; VEGF: Forward Sequence TTGCCTTGCTGCTCTACCTCCA, Reverse Sequence GATGGCAGTAGCTGCGCTGATA; β-actin: Forward Sequence CACCATTGGCAATGAGCGGTTC, Reverse Sequence AGGTCTTTGCGGATGTCCACGT.

### 2.4 Chemiluminescent Enzyme Immunoassay (CLEIA)

CLEIA assay was performed using CLEIA assay kit for VEGF (Weigao Biotech Co., Ltd. Weihai, Shandong, China) in accordance with the manufacturer's instructions. The culture supernatants of the cells, HRP conjugated detection antibody, were, respectively, added into the wells of the test plate that had been previously coated with anti-human VEGF antibody, incubated for 1 h at 37°C and washed 5 times. After incubation with the chemiluminescent substrate reagents (solution A and B) in the dark, the test plate was read immediately on a CLEIA system. The concentration of VEGF was determined by calculation with standard curve equation of known VEGF concentrations.

### 2.5 CCK-8 Assay

Cell proliferation was measured by CCK-8 assay in accordance with the manufacturer's instructions. The HUVECs were seeded into 96 well plate at 5×10^3^ cells/well for overnight culture and the medium in the wells was replaced by conditioned medium (200 µl/well) of the Erbin over-expression SKBR3 cells and Erbin silence MCF-7 cells collected 36 hour after their transfection. The cells were detected using CCK-8 at 0 hour, 6 hour, 12 hour and 24 hour after replacement by the conditioned medium and read at 450 nm on a microplate reader. The cell proliferation was calculated based on the absorbance values.

Proliferation (%) = (OD_test hour_ / OD_NC 0 hour_) × 100%.

### 2.6 Angiogenic Matrigel Tube Formation Assay

^®^The Matrigel Tube Formation Assay was used to detect the angiogenic ability of HUVECs *in vitro*. The Matrigel Matrix (Corning, Tewksbury, MA, USA) thawed at 4°C overnight was mixed with equal volume of DMEM medium and was added to 96-well plates (50 μl/well) followed by 1h polymerization at 37°C for formation of a thin gel layer. The HUVECs in the conditioned medium were seeded into 96-well plates containing Matrigel (3×10^4^ cells/well). After the cells were cultured for 4h, the picture of the capillary-like structures was captured with a CCD under a light microscope and analyzed using the Angiogenesis Analyzer plug-in for ImageJ software (NIH, USA).

### 2.7 Statistical Analysis

The data were shown as mean ± standard deviation (SD) and statistical significance was determine using t test. Three independent experiments were performed. *p* < 0.05 was statistically considered significant.

## 3 RESULTS

### 3.1 The Erbin Expression was Lower in SKBR3 Cells but Higher in MCF-7 Cells and was Over-expressed by Erbin Gene Transfection in SKBR3 Cells and Silenced by Erbin siRNA Transfection in MCF-7 Cells

**Fig. (1):**
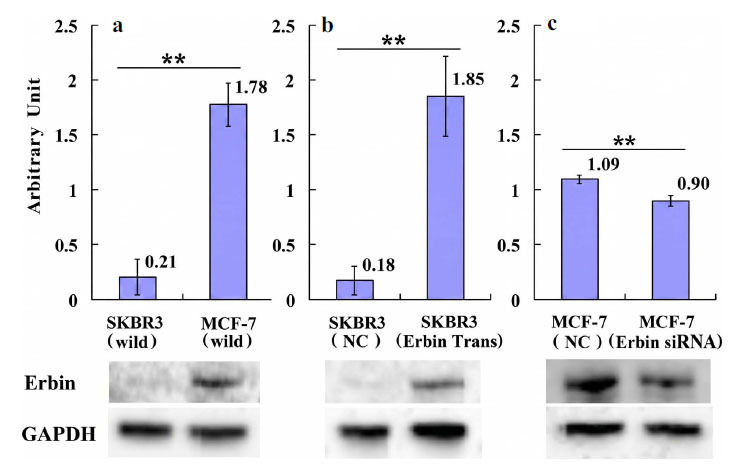
The expression of Erbin protein in the cells. The expression of the Erbin protein in wild SKBR3 cells and wild MCF-7 cells (**a**), in SKBR3 cells transfected with the plasmids encoding the full-length Erbin gene (**b**) and MCF-7 cells transfected with the Erbin siRNA (**c**) was detected by Western blot. The non-targeting controls (NC) for the plasmids and siRNA were used, respectively. Double asterisk indicates *p*-values < 0.01, compared with the control after t test.

**Fig. (2):**
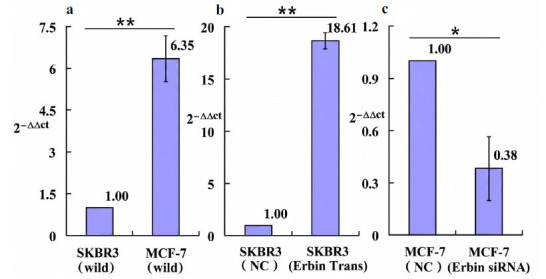
The expression of Erbin mRNA in the cells. The expression of the Erbin mRNA in wild SKBR3 cells and wild MCF-7 cells (**a**), in SKBR3 cells transfected with the plasmids encoding the full-length Erbin gene (**b**) and MCF-7 cells transfected with the Erbin siRNA (**c**) was detected by qRT-PCR. The non-targeting controls (NC) for the plasmids and siRNA were used, respectively. Asterisk indicates *p*-values < 0.05, and double asterisk indicates *p*-values < 0.01, compared with the control after t test.

In breast cancer, the human epidermal growth factor receptor 2 (HER2, also named ERBB2) positive subtype is historically related to a particularly poor prognosis [52]. SKBR3 cell line and MCF-7 cell line are weakly invasive breast cancer cell lines, while SKBR3 cell line is ER−/ERBB2+ ‘weakly luminal epithelial-like’ and MCF-7 cell line is ER+/ERBB2− ‘luminal epithelial-like’ [53]. The expression of Erbin in SKBR3 cells and MCF-7 cells is considerable because Erbin is closely associated with HER2. It was shown that the expression of the Erbin protein in SKBR3 cells was lower than that in MCF-7 cells with statistical significance (*p* < 0.01) detected by western blot (Fig. **1a**). The Erbin mRNA in SKBR3 cells was lower than that in MCF-7 cells with statistical significance (*p* < 0.01) detected by qRT-PCR (Fig. **2a**).

The expression of the Erbin protein in SKBR3 cells was successfully over-expressed by Erbin gene transfection compared with the NC SKBR3 cells (Fig. **1b**) and silenced in MCF-7 cells by Erbin siRNA transfection compared with the NC MCF-7 cells (Fig. **1c**), with statistical significance (*p* < 0.01), detected by western blot. The Erbin mRNA in SKBR3 cells was over-expressed by Erbin gene transfection (*p* < 0.01) compared with the NC SKBR3 cells (Fig. **2b**) and silenced in MCF-7 cells by Erbin siRNA transfection (*p* < 0.05) compared with the NC MCF-7 cells (Fig. **2c**), with statistical significance (*p* < 0.05), detected by qRT-PCR.

### 3.2 The Expression of VEGF was Higher in SKBR3 Cells and Lower in MCF-7 Cells but was Inhibited by Transfection with Erbin Gene in SKBR3 Cells and Promoted by Transfection with Erbin siRNA in MCF-7 Cells

**Fig. (3):**
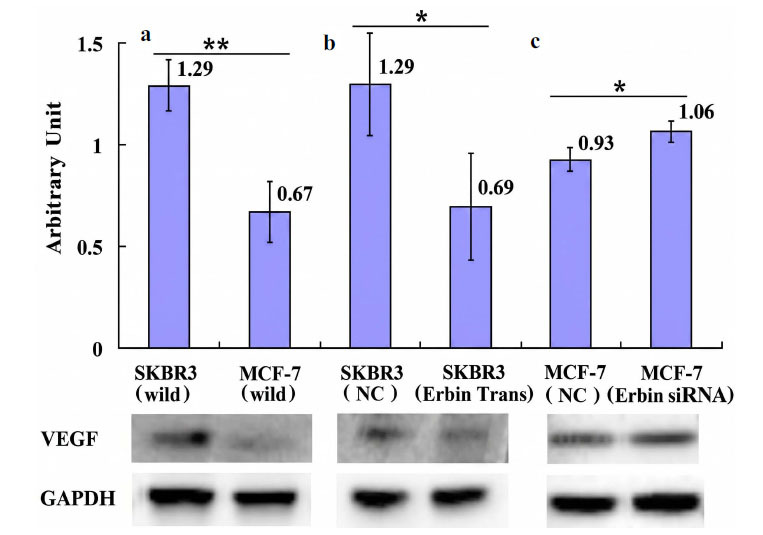
The expression of VEGF protein in the cells. The expression of the VEGF protein in wild SKBR3 cells and wild MCF-7 cells (**a**), in SKBR3 cells transfected with the plasmids encoding the full-length Erbin gene (**b**) and MCF-7 cells transfected with the Erbin siRNA (**c**) was detected by Western blot. The non-targeting controls (NC) for the plasmids and siRNA were used, respectively. Asterisk indicates *p*-values < 0.05, and double asterisk indicates *p*-values < 0.01, compared with the control after t test.

VEGF is one of the most important angiogenic factors produced and released by cancer cells, including breast cancer cells, to induce tumor angiogenesis for nutrient and oxygen supply and metabolite waste removal. It was shown that the expression of VEGF protein was higher in SKBR3 cells than that in MCF-7 cells with statistical significance (*p* < 0.01) detected by western blot (Fig. **3a**).

**Fig. (4):**
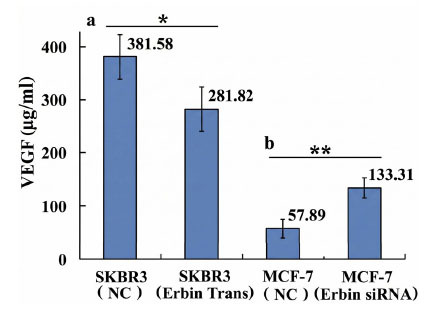
The expression of VEGF protein in the culture supernatants of the cells. The expression of the VEGF protein in the culture supernatants of SKBR3 cells transfected with the plasmids encoding the full-length Erbin gene (**a**) And MCF-7 cells transfected with the Erbin siRNA (**b**) Was detected by Chemiluminescent Enzyme Immunoassay (CLEIA). The non-targeting controls (NC) for the plasmids and siRNA were used, respectively. Asterisk indicates *p*-values < 0.05, and double asterisk indicates *p*-values < 0.01, compared with the control after t test.

**Fig. (5):**
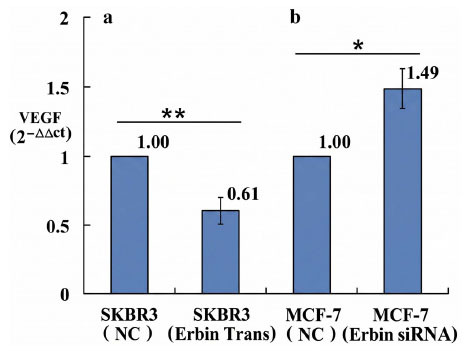
The expression of VEGF mRNA in the cells. The expression of the VEGF mRNA in SKBR3 cells transfected with the plasmids encoding the full-length Erbin gene (**a**) and MCF-7 cells transfected with the Erbin siRNA (**b**) was detected by qRT-PCR. The non-targeting controls (NC) for the plasmids and siRNA were used, respectively. Asterisk indicates *p*-values < 0.05, and double asterisk indicates *p*-values < 0.01, compared with the control after t test.

With the over-expression of Erbin by transfection of Erbin gene in SKBR3 cells, the expression of VEGF protein was downregulated compared with the NC SKBR3 cells (Fig. **3b**) and upregulated with the Erbin silence by transfection of Erbin siRNA in MCF-7 cells compared with the NC MCF-7 cells (Fig. **3c**), with statistical significance (*p* < 0.05) detected by western blot. The release of VEGF into the cell culture supernatant was downregulated with the over-expression of Erbin by transfection of Erbin gene in SKBR3 cells (*p* < 0.05), compared with the NC SKBR3 cells (Fig. **4a**) and upregulated with the Erbin silence by transfection of Erbin siRNA in MCF-7 cells (*p* < 0.01) compared with the NC MCF-7 cells, with statistical significance (*p* < 0.05) detected by ELISA (Fig. **4b**). The VEGF mRNA was downregulated with the over-expression of Erbin by transfection of Erbin gene in SKBR3 cells (*p* < 0.01), compared with the NC SKBR3 cells (Fig. **5a**) and upregulated with the Erbin silence by transfection of Erbin siRNA in MCF-7 cells (*p* < 0.05) compared with the NC MCF-7 cells (Fig. **5b**), with statistical significance (*p* < 0.05) detected by qRT-PCR.

### 3.3 Conditioned Medium from Erbin Over-expression SKBR3 Cells Downregulated HUVEC Proliferation and Conditioned Medium from Erbin Silencing MCF-7 Cells Upregulated HUVEC Proliferation

**Fig. (6):**
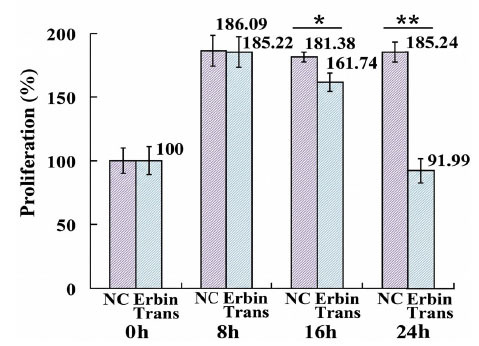
The proliferation of HUVECs in the conditioned medium of SKBR3 cells. The proliferation of HUVECs in the conditioned medium of SKBR3 cells transfected with the plasmids encoding the full-length Erbin gene was detected by CCK8 assay. The non-targeting control (NC) for the plasmids was used, respectively. Asterisk indicates *p*-values < 0.05, and double asterisk indicates *p*-values < 0.01, compared with the control after t test. Proliferation (%) = (OD_test hour_ / OD_NC 0 hour_) × 100%.

**Fig. (7):**
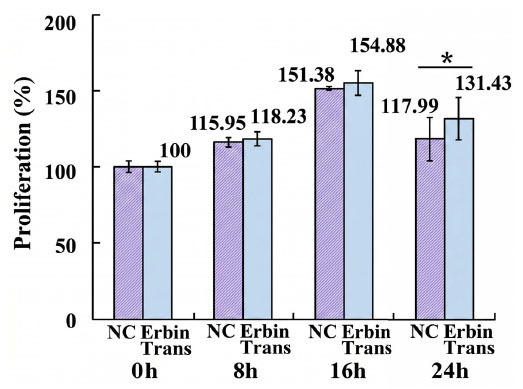
The proliferation of HUVECs in the conditioned medium of MCF-7 cells. The proliferation of HUVECs in the conditioned medium of MCF-7 cells transfected with the Erbin siRNA was detected by CCK8 assay. The non-targeting control (NC) for the plasmids was used, respectively. Asterisk indicates *p*-values < 0.05, compared with the control after paired t test. Proliferation (%) = (OD_test hour_ / OD_NC 0 hour_) × 100%.

It was shown that, with the over-expression of Erbin by transfection of Erbin gene in SKBR3 cells, the proliferation of HUVEC in the conditioned medium was downregulated at 16h (*p* < 0.05) and 24h (*p* < 0.01) compared with that in the conditioned medium from NC SKBR3 cells (Fig. **6**) and upregulated at 24h (*p* < 0.05) in the conditioned medium from MCF-7 cells with the Erbin silence by transfection of Erbin siRNA compared with that in the conditioned medium from NC MCF-7 cells (Fig. **7**), with statistical significance (*p* < 0.05) detected by CCK-8.

### 3.4 Conditioned Medium from the Erbin Over-expression SKBR3 Cells induced Less Angiogenic Effect than that from NC SKBR3 Cells but Conditioned Medium from Erbin Silence MCF-7 Cells Induced more Angiogenic Effect than that from NC MCF-7 Cells

**Fig. (8):**
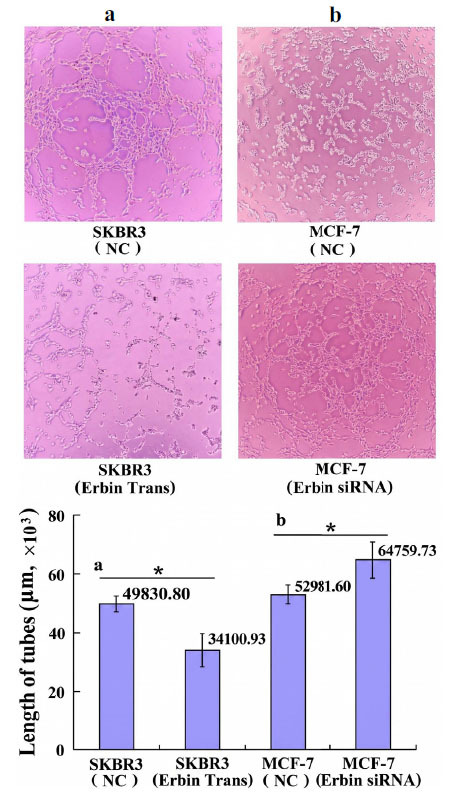
The tube formation of HUVECs in the conditioned medium of the cells. The tube formation of HUVECs in the conditioned medium of SKBR3 cells transfected with the plasmids encoding the full-length Erbin gene (**a**) and MCF-7 cells transfected with the Erbin siRNA (**b**) was detected by Angiogenic Matrigel tube formation assay. The total length of tube formation in the conditioned medium was shown (Magnification, ×100) under an Echo RVL2-k2 Revolve Fluorescence Touchscreen Microscope (Echo, california, USA). The non-targeting controls (NC) for the plasmids and siRNA were used, respectively. Asterisk indicates *p*-values < 0.05, compared with the control after t test.

With the quantification using ImageJ software, the total length of the tube was determined in tube formation assay. As shown in Fig. (**8**), with the over-expression of Erbin by transfection of Erbin gene in SKBR3 cells, the proangiogenic effect in the conditioned medium was downregulated compared with that in the conditioned medium from NC SKBR3 cells (Fig. **8a**) and upregulated in the conditioned medium from MCF-7 cells with the Erbin silence by transfection of Erbin siRNA compared with that in the conditioned medium from NC MCF-7 cells (Fig. **8b**), with statistical significance (*p* < 0.05) .

### 3.5 The Expression of pSTAT3 was Downregulated by Transfection with Erbin Gene in SKBR3 Cells and Upregulated by Transfection with Erbin siRNA in MCF-7 Cells

**Fig. (9):**
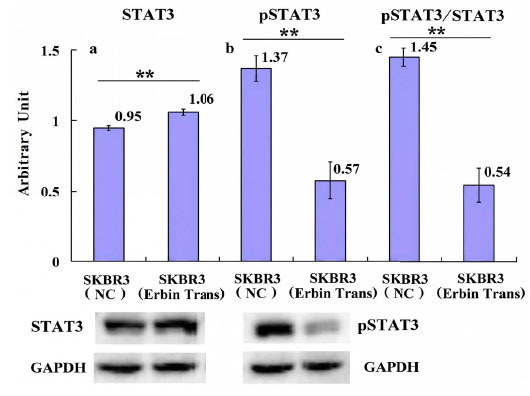
The expression of STAT3 and pSTAT3 proteins in SKBR3 cells. The expression of the STAT3 (**a**) and pSTAT3 (**b**) proteins in SKBR3 cells transfected with the plasmids encoding the full-length Erbin gene was detected by Western blot (**c**). The non-targeting controls (NC) for the plasmids were used. Double asterisk indicates *p*-values < 0.01, compared with the control after t test.

With the over-expression of Erbin by transfection of Erbin gene in SKBR3 cells, the expression of pSTAT3 protein was downregulated compared with the NC SKBR3 cells (Fig. **9**) with statistical significance (*p* < 0.01), and the expression of pSTAT3 protein with the Erbin silence by transfection of Erbin siRNA in MCF-7 cells compared with the NC MCF-7 cells was shown in Fig. (**10**), detected by Western blot.

## 4 DISCUSSION

Erbin is a cytoplasmic protein expressed in mouse mammary epithelial cells and is associated with ErbB2-dependent proliferation in breast cancer cells [54]. Erbin contains leucine-rich repeats (LRR) and PSD95/Dlg1/zo-1 (PDZ) domain (thus named a LAP protein) [55, 56] to interact specifically with ErbB2 instead of ErbB3, ErbB4, or EGFR, *via* its single PDZ domain [57]. Erbin is an inhibitor in diverse signal pathways and its downregulation accelerates cell migration and induces trastuzumab resistance in Her2-overexpressing breast cancer cells [58-61]. The inhibitory role of Erbin in tumor angiogenesis, however, remains to be demonstrated. In this study, using conditioned medium from SKBR3 and MCF-7 breast cancer cells, the inhibitory role of Erbin in HUVEC proliferation and angiogenic effect was demonstrated.

Positive Her2 in SKBR3 cells [62-64] but negative in MCF-7 cells [65-67] and its close association with Erbin in breast cancer cells [58] indicate the possibility of different expression of Erbin in these two cell lines. As shown in this study, the lower expression of Erbin in SKBR3 cells but higher in MCF-7 cells makes the two cell lines may be used to examine the roles of Erbin in biological processes including angiogenesis. The Erbin was over-expressed in SKBR3 cells by Erbin gene transfection while silenced in MCF-7 cells by Erbin siRNA transfection in this study to examine the roles of Erbin variation in angiogenesis.

HUVEC proliferation is important in angiogenesis [68-71]. The CCK-8 assay and the Matrigel Tube Formation Assay demonstrated, respectively, that the HUVEC proliferation and angiogenic effects was downregulated in conditioned medium from Erbin over-expression SKBR3 cells and upregulated in conditioned medium from Erbin silence MCF-7 cells. These results demonstrated that Erbin in breast cancer cells inhibited HUVEC proliferation and angiogenesis *in vitro*.

VEGF is the most common and strongest elicitor of the various factors inducing significant angiogenic response [72-75]. It was shown in this study that the level of VEGF was higher in SKBR3 cells and in the culture supernatant of SKBR3 cells than that in MCF-7 cells and in the culture supernatant of MCF-7 cells, respectively, but was downregulated in Erbin over-expression SKBR3 cells and upregulated in Erbin silence MCF-7 cells. The levels of VEGF in the conditioned media were corresponding with their effects on HUVEC proliferation and angiogenesis *in vitro*. The fact that VEGF is regulated by STAT3 dependent pathway [76] implies that the effects of the conditioned media on HUVEC proliferation and angiogenesis may be regulated by STAT3 dependent pathway. Therefore, the expression of pSTAT3 was detected showing that the expression of pSTAT3 was downregulated in SKBR3 cells with over-expression of Erbin by transfection with Erbin gene and upregulated in MCF-7 cells with Erbin silence by transfection with Erbin siRNA. These results were in line with the levels of VEGF in the conditioned media and their effects on HUVEC proliferation and angiogenesis *in vitro*.

## CONCLUSION

In conclusion, with the over-expression and silence of Erbin gene, the VEGF in the cells and in the conditioned medium, HUVEC proliferation and angiogenesis in the conditioned medium, and pSTAT3 protein in the cells, were downregulated by transfection of Erbin gene in SKBR3 cells and upregulated by transfection of Erbin siRNA in MCF-7 cells, indicating that, with the inhibition of the STAT3 pathway in the breast cancer cells, Erbin inhibited the proangiogenic effect of breast cancer cells with potential to target Erbin for breast cancer therapy.

## Figures and Tables

**Fig. (10):**
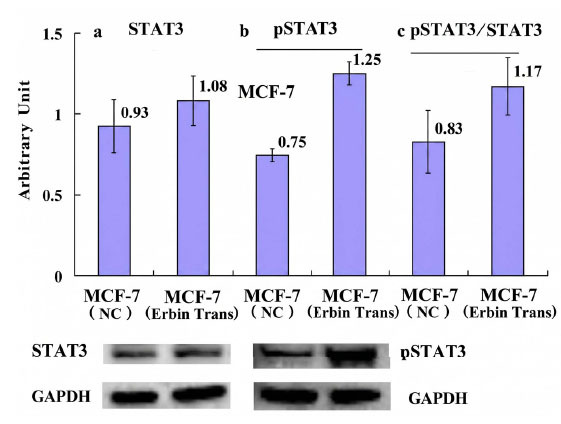
The expression of STAT3 and pSTAT3 proteins in MCF-7 cells. The expression of the STAT3 (**a**) and pSTAT3 (**b**) proteins in MCF-7 cells transfected with the Erbin siRNA was detected by Western blot. (**c**) The non-targeting controls (NC) for the plasmids were used. Double asterisk indicates *p*-values < 0.01, compared with the control after t test.

## Data Availability

All the data generated or analyzed during this study are included in this published article.

## LIST OF ABBREVIATIONS

EPCs: Endothelial Progenitor Cells
VEGF: Vascular Endothelial Growth Factor
bFGF: Basic Fibroblast Growth Factor
PDGF: Platelet-derived Growth Factor
